# Habitat-Mediated Spillover Risk of Andes Hantavirus in *Oligoryzomys longicaudatus*: A Mechanistic Eco-Epidemiological Model

**DOI:** 10.3390/v18091024

**Published:** 2026-09-15

**Authors:** Hendrik Sulbaran-Pineda, Fernando Córdova-Lepe, Luis Pastenes, Juan Pablo Gutiérrez-Jara, Beatriz Cancino-Faure

**Affiliations:** 1 Facultad de Ciencias Básicas, Universidad Católica del Maule, Talca 3480112, Chile; lpastenes@ucm.cl; 2 Centro de Ecología, Instituto Venezolano de Investigaciones Científicas, Altos de Pipe 1204, Miranda, Venezuela; 3 Centro de Investigación de Estudios Avanzados del Maule, Universidad Católica del Maule, Talca 3480112, Chile; jgutierrez@ucm.cl; 4 Facultad de Medicina, Universidad Católica del Maule, Talca 3480112, Chile; bcancino@ucm.cl

**Keywords:** andes hantavirus, rodent-borne viruses, *Oligoryzomys longicaudatus*, reservoir ecology, spillover risk, habitat degradation, ecological restoration, One Health surveillance, effective reproduction number, eco-epidemiological modeling

## Abstract

Andes hantavirus (ANDV) is a rodent-borne orthohantavirus associated with hantavirus cardiopulmonary syndrome in southern South America. Its maintenance and spillover risk depend on the ecology of its principal reservoir, *Oligoryzomys longicaudatus*, and on environmental changes that alter habitat availability, host abundance, and human–rodent interfaces. We developed a mechanistic eco-epidemiological model that couples effective habitat cover to an SIR framework for ANDV transmission in *O. longicaudatus*. Habitat degradation and compensatory restoration modify rodent carrying capacity, natality, and the force of infection, which depends on infected host load relative to instantaneous ecological capacity. We derived the habitat equilibrium, the basic reproduction number $R_{0}$, and the time-dependent effective reproduction number $R_{e}\left(t\right)$ and evaluated infection-burden and threshold indicators across degradation-restoration scenarios. The analysis shows that $R_{0}$ is independent of equilibrium habitat cover because susceptible abundance scales with carrying capacity at the disease-free equilibrium. In contrast, $R_{e}\left(t\right)$, cumulative incidence, and infected load depend on transient habitat-mediated crowding. Restoration increases reservoir abundance and absolute infection burden, whereas degradation can reduce abundance while increasing crowding-driven transmission pressure and prolonging supercritical windows. These results identify ecological conditions under which habitat change may intensify reservoir infection pressure and guide One Health surveillance at human–rodent interfaces.

## 1. Introduction

Anthropogenic disturbance can alter contact rates among wildlife reservoirs, vectors, domestic animals, and humans, thereby reshaping zoonotic emergence risk [1,2,3,4]. Deforestation and habitat fragmentation have received particular attention because disturbed landscapes can favor pathogens associated with wildlife reservoirs [5]. These disturbances can benefit generalist reservoirs with broad distributions, synanthropic behavior, and high ecological resilience [6].

Rodents are central reservoirs for many zoonotic pathogens transmitted by direct contact, environmental contamination, or spillover to related host species [7,8,9]. The genus *Orthohantavirus*, within the family *Hantaviridae*, provides a clear example: its main reservoirs belong to Muridae, including the subfamilies Murinae, Arvicolinae, and Sigmodontinae [10]. Hantavirus infection in rodents is usually persistent and asymptomatic, and infected hosts can shed virus in urine, feces, and saliva [11]. Humans acquire infection primarily by inhaling aerosolized excreta, although bites or direct contact may also transmit virus [12]. The main clinical outcome in the Americas is hantavirus cardiopulmonary syndrome, which has high case-fatality and therefore strong public-health relevance [13,14]. Among hantaviruses, ANDV is unusual in its demonstrated capacity for sustained person-to-person transmission, which has direct implications for infection control and hospital isolation [15].

Human-modified habitats can favor transmission and persistence, but the mechanisms remain difficult to separate [16]. Disturbance may increase contact rates, reduce habitat availability, change resource distribution, or restructure host communities. In rodent-borne systems, these processes can alter host abundance, local crowding, dispersal, and contact with humans. A mechanistic model should therefore distinguish demographic effects of habitat from transmission effects of habitat.

Empirical studies support a link between habitat reduction and increased transmission in rodent reservoirs. Forced aggregation can elevate pathogen prevalence [17]; fragmentation can increase Sin Nombre virus infection rates [18,19]; and reservoir density can modulate hantavirus transmission [20,21].

Most hantavirus models emphasize reservoir population dynamics without representing habitat as a dynamic state variable [22,23,24,25,26]. Multispecies epizootic models for susceptible and infected hosts have also been proposed [27], but many do not separate habitat loss, demographic regulation, and transmission crowding. This gap limits the ability of current models to evaluate restoration and degradation scenarios as explicit management interventions.

Models can represent habitat implicitly, by embedding habitat effects in vital rates, or explicitly, by defining habitat variables that enter demographic and epidemiological equations. Explicit representations are preferable for scenario analysis because the habitat state can be perturbed directly and its effects can propagate through population growth, carrying capacity, and transmission [28].

We developed, analyzed, and simulated an eco-epidemiological model for intraspecific ANDV transmission in *Oligoryzomys longicaudatus*, the main hantavirus reservoir in Argentina and Chile [29,30]. Active ANDV circulation in this reservoir remains a concern, as evidenced by the recent isolation of a novel viral haplotype from a human cluster in Chile [31]. *O. longicaudatus* is an opportunistic generalist with broad ecological plasticity, occurring from humid temperate forests to steppe ecotones and reaching high abundance in dense understory vegetation with shrub cover [7,29]. Its abundance can increase after bamboo flowering, crop pulses, or landscape disturbance, making habitat-mediated demography and contact structure central to ANDV transmission dynamics [7,29,32].

Accordingly, the model treats habitat cover as a dynamic ecological state that regulates reservoir carrying capacity, natality, and transmission pressure. By linking habitat degradation, compensatory restoration, reservoir abundance, infected load, and the time-dependent effective reproduction number $R_{e}\left(t\right)$, the framework identifies ecological conditions that may increase viral persistence in the reservoir and intensify exposure risk at human–rodent interfaces. The study addresses three questions: how habitat change alters infection burden in the reservoir, how degradation and restoration reshape transient transmission risk, and how habitat-mediated crowding can create surveillance-relevant windows of increased spillover potential.

## 2. Materials and Methods

This section defines the eco-epidemiological model, the biological assumptions, and the threshold quantities used in the numerical analysis of reservoir infection pressure. The model couples a scalar habitat equation to an SIR (susceptible–infected–removed) system for the rodent reservoir. Table 1 and Table 2 summarize the state variables and parameters.

### 2.1. Eco-Epidemiological Model Structure and Effective Habitat Dynamics

**Figure 1:**
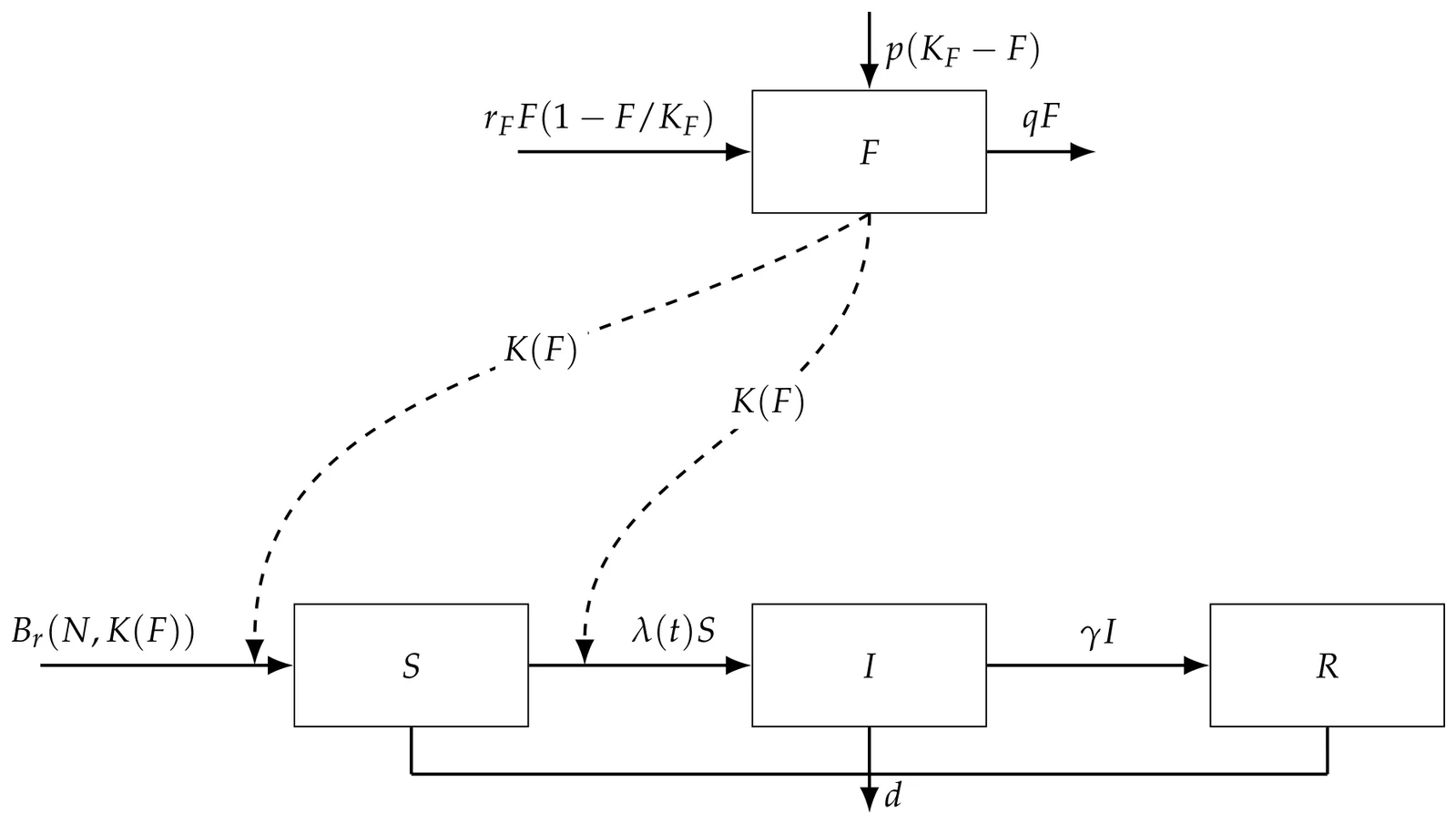
Schematic diagram of the coupled eco-epidemiological model. Solid arrows indicate demographic and epidemiological flows; dashed arrows indicate that effective habitat *F* determines carrying capacity K(F), which regulates density-dependent natality and the force of infection. *S*, *I*, *R*: susceptible, infected, and removed rodents; $B_{r}\left(N,K\left(F\right)\right)$: density-dependent natality; $\lambda \left(t\right)=\beta_{0}I/K\left(F\right)$: force of infection, with $\beta_{0}$ the baseline effective transmission coefficient; γ: removal rate; *d*: natural mortality rate; $r_{F}$: intrinsic habitat regeneration rate; $K_{F}$: maximum effective habitat; p=αq and *q*: restoration and degradation rates.

Let F(t) denote the effective usable habitat available to *Oligoryzomys longicaudatus* in a focal landscape. Figure 1 presents the overall structure of the coupled eco-epidemiological model.The variable *F* represents an area-equivalent measure of shelter, nesting sites, understory cover, and foraging substrate. We assume $0<F\left(t\right)\leq K_{F}$, where $K_{F}$ is the maximum effective habitat level in the focal landscape. This reduction of spatially heterogeneous habitat attributes to a scalar state variable follows aggregation principles in mathematical ecology and landscape models that represent territorial or environmental capacity through ordinary differential Equations [33,34,35,36].

Habitat changes through natural regeneration, anthropogenic degradation, and compensatory restoration. Natural regeneration follows logistic growth with intrinsic rate $r_{F}>0$ and upper bound $K_{F}$. Degradation acts on available habitat at rate q≥0. Restoration acts on the habitat deficit $K_{F}-F$ at rate p=αq, where α∈[0,1] is the compensation coefficient. Thus α=0 represents no restoration, whereas α=1 represents full compensation of the imposed degradation rate. The habitat Equation is

(1) $$\frac{dF}{dt}=r_{F}F\left(1-\frac{F}{K_{F}}\right)+p\left(K_{F}-F\right)-qF,\;p=\alpha q.$$

In this study, *p* and *q* are constant scenario parameters. Time-dependent degradation or restoration functions are possible extensions, but they are not used in the threshold analysis or numerical simulations.

Setting $\dot{F}=0$ in (1) gives

(2) $$-\frac{r_{F}}{K_{F}}{\left(F^{*}\right)}^{2}+\left(r_{F}-p-q\right)F^{*}+pK_{F}=0.$$

The biologically relevant nonnegative root is

(3) $$F^{*}=\frac{K_{F}}{2r_{F}}\left[\left(r_{F}-p-q\right)+\sqrt{{\left(r_{F}-p-q\right)}^{2}+4r_{F}p}\right].$$

If p>0, this root is positive for all q≥0. If p=0, it reduces to $F^{*}=K_{F}\left(1-q/r_{F}\right)$ and is positive only when $r_{F}>q$. Thus active restoration prevents collapse to F=0 in this scalar habitat equation, whereas the no-restoration boundary case requires $r_{F}>q$.

Rodent carrying capacity depends on habitat according to

(4) $$K\left(F\right)=\frac{K_{r}}{K_{F}}F,$$

where $K_{r}$ is the maximum rodent carrying capacity when $F=K_{F}$.

### 2.2. Rodent Demography and ANDV Transmission Dynamics

We focus on *O. longicaudatus* because its ecology supports a habitat-mediated mechanism for ANDV persistence. This sigmodontine rodent uses dense understory, shrub cover, forest edges, and rural-peridomestic ecotones for shelter and foraging [7,29,32]. Changes in vegetation cover can therefore alter carrying capacity, local movement, and contact structure. These features motivate a model in which effective habitat regulates both natality and transmission.

The rodent population is divided into susceptible *S*, infected *I*, and removed *R* classes, with total abundance N=S+I+R. The model describes intraspecific transmission within the reservoir population; human spillover is interpreted indirectly through ecological indicators of reservoir infection pressure and exposure potential. The subscript *r* denotes the rodent reservoir. Natality follows density-dependent regulation, a standard assumption in rodent hantavirus models that links population growth to carrying capacity and resource limitation [37,38]:

(5) $$B_{r}\left(N,K\left(F\right)\right)=b_{r}N{\left(1-\frac{N}{K(F)}\right)}_{+},$$

where $b_{r}$ is the intrinsic birth rate and ${\left(x\right)}_{+}=\max\left\{x,0\right\}$. The positive-part operator prevents negative births when abrupt habitat contraction produces transient states with N>K(F).

The force of infection is

(6) $$\lambda \left(t\right)=\beta_{0}\frac{I(t)}{K(F(t))},$$

where I(t)/K(F(t)) represents infected host load relative to instantaneous ecological carrying capacity [39]. This form follows density-dependent transmission logic: infection risk increases with the concentration of infectious hosts in the space effectively occupied by the population. In this model, K(F) represents available habitat through ecological capacity; therefore, habitat contraction lowers capacity and concentrates infectious rodents in the remaining usable habitat.

This formulation is consistent with disease-ecology frameworks in which rodent-borne zoonotic risk depends on reservoir abundance, infected-host density, environmental deposition of pathogens, and with landscape-conversion models in which habitat loss changes carrying capacity, local density, and contact opportunities [40,41]. We interpret I/K(F) as a crowding-load term, not as epidemiological prevalence. Regularized or saturated transmission functions may be more appropriate when data show lower bounds in habitat use, contact avoidance, or saturation in contact rates. We retain the linear inverse form because it isolates the habitat-crowding mechanism and keeps the threshold analysis tractable.

We emphasize that $\beta_{0}$ is an effective transmission coefficient rather than a purely direct-contact rate. It aggregates, at a fixed environmental baseline, both direct rodent-to-rodent transmission and the indirect route mediated by environmental deposition of virus in excreta, nests, and contaminated substrate. This lumped representation is standard in host–microparasite formulations that distinguish transmission mechanisms while retaining a single incidence coefficient [39]. We do not introduce a separate environmental compartment for viral persistence because an explicit indirect-transmission structure requires shedding, environmental clearance, and indirect-contact parameters that, in the models where they appear, are identifiable only through calibration to longitudinal case or contamination series [26,42]. Because the present study is a threshold and scenario analysis that is deliberately not fitted to seroprevalence or environmental data, embedding such parameters would reduce the transparency and identifiability of the model. We therefore retain the indirect route implicitly within $\beta_{0}$ and treat an explicit environmental compartment as a calibration-dependent extension.

The SIR subsystem is

(7) $$\begin{array}{c} \left\{\begin{array}{cc} \frac{dS}{dt} & =B_{r}\left(N,K\left(F\right)\right)-dS-\lambda \left(t\right)S,\\ \frac{dI}{dt} & =\lambda (t)S-(d+\gamma )I,\\ \frac{dR}{dt} & =\gamma I-dR. \end{array}\right. \end{array}$$

Here *d* is the natural mortality rate, and γ is the removal rate. We assume no vertical transmission, no disease-induced mortality, and a removed class that represents seropositive individuals with negligible or reduced shedding, consistent with chronic low-pathogenicity hantavirus infection in reservoir hosts [23,32,43,44]. In Chile, the long-term serological surveillance of *O. longicaudatus* supports this persistent, largely asymptomatic infection profile in the reservoir [32].

The complete system is

(8) $$\begin{array}{c} \left\{\begin{array}{cc} \frac{dF}{dt} & =r_{F}F\left(1-\frac{F}{K_{F}}\right)+p\left(K_{F}-F\right)-qF,\;p=\alpha q,\\ \frac{dS}{dt} & =B_{r}\left(N,K\left(F\right)\right)-dS-\beta_{0}\frac{SI}{K(F)},\\ \frac{dI}{dt} & =\beta_{0}\frac{SI}{K(F)}-\left(d+\gamma \right)I,\\ \frac{dR}{dt} & =\gamma I-dR. \end{array}\right. \end{array}$$

**Table 2:** Model parameters. Time is measured in months. Baseline values and ranges are biologically plausible for *O. longicaudatus* ecology and landscape management scenarios. Units: [ind] denotes individual rodents and [ha] denotes hectares.

Parameter	Description	Base	Range	Unit	Source
$\beta_{0}$	Baseline effective transmission coefficient (contact frequency × transmission probability), combining direct contact and indirect exposure to contaminated substrate.	0.45	0.10–1.00	[month^−1^]	[37,42]
γ	Removal rate.	0.20	0.00–0.30	[month^−1^]	[37,42,44,45]
$b_{r}$	Intrinsic birth rate.	0.30	0.10–0.60	[month^−1^]	[7,37,46]
*d*	Natural mortality rate.	0.10	0.05–0.30	[month^−1^]	[7,37,46]
$K_{r}$	Maximum rodent carrying capacity at $F=K_{F}$.	20	5–50	[ind]	[7,38,46]
$r_{F}$	Intrinsic habitat regeneration rate.	0.001	0.001–0.005	[month^−1^]	[47,48]
*q*	Anthropogenic degradation rate.	0.010	0.0004–0.019	[month^−1^]	[47,48]
α	Compensation coefficient defining restoration as p=αq.	0.40	0.00–1.00	[–]	this work
$K_{F}$	Maximum effective habitat level, base on home-range studies of *O. longicaudatus*.	1.0	0.4–3.7	[ha]	[49]

### 2.3. Analytical Properties and Threshold Quantities

For nonnegative initial conditions with $0<F\left(0\right)\leq K_{F}$, the biologically feasible region is positively invariant: $0<F\left(t\right)\leq K_{F}$, S(t),I(t),R(t)≥0, and N(t) remains bounded. Hence K(F(t))>0 along biologically admissible trajectories, and the force of infection is well defined. Appendix A gives the proof.

A positive rodent demographic equilibrium requires $b_{r}>d$, because births must exceed natural mortality at low density. Under this feasibility condition, the disease-free equilibrium is

(9) $$E_{0}=\left(F^{*},S_{0}^{*},0,0\right),\;S_{0}^{*}=K\left(F^{*}\right)\left(1-\frac{d}{b_{r}}\right).$$

The basic reproduction number is

(10) $$R_{0}=\frac{\beta_{0}}{d+\gamma }\left(1-\frac{d}{b_{r}}\right).$$

This expression does not depend on $F^{*}$ because susceptible abundance scales with $K(F^{*})$ at the disease-free equilibrium. Thus habitat-dependent carrying capacity cancels in the equilibrium invasion threshold.

Along trajectories, the effective reproduction number is

(11) $$R_{e}\left(t\right)=\frac{\beta_{0}}{d+\gamma }\frac{S(t)}{K(F(t))}.$$

Habitat affects transmission through S(t)/K(F(t)), which controls $R_{e}\left(t\right)$ and the incidence term. Thus degradation and restoration can modify transient transmission, infected load, and the duration of supercritical periods even though $R_{0}$ does not depend on $F^{*}$.

When $R_{0}>1$, the endemic equilibrium is

(12) $$E_{1}=\left(F^{*},S_{1}^{*},I_{1}^{*},R_{1}^{*}\right),$$

with

(13) $$S_{1}^{*}=\frac{\left(d+\gamma \right)K\left(F^{*}\right)}{\beta_{0}},\;I_{1}^{*}=\frac{d}{d+\gamma }\left[K\left(F^{*}\right)\left(1-\frac{d}{b_{r}}\right)-S_{1}^{*}\right],\;R_{1}^{*}=\frac{\gamma }{d}I_{1}^{*}.$$

The disease-free equilibrium is locally asymptotically stable when $R_{0}<1$ and unstable when $R_{0}>1$. For $R_{0}>1$, the endemic equilibrium exists uniquely. The threshold at $R_{0}=1$ corresponds to a forward transcritical bifurcation. Table 3 summarizes the equilibria and threshold conditions of the system. Appendix B and Appendix C provide the derivations.

### 2.4. Epidemiological Metrics and Threshold Indicators

Let $x\left(t\right)={\left[F,S,I,R\right]}^{⊤}$ denote the solution of system (8) over $t\in [0,t_{f}]$. We use two sets of quantities. The first summarizes infection burden over the full horizon $\left[0,t_{f}\right]$. The second summarizes threshold behavior over the final analysis window $\left[t_{*},t_{f}\right]$ after discarding transients.

The instantaneous incidence rate is

(14) $$I\left(t\right)=\beta_{0}\frac{S(t)I(t)}{K(F(t))}.$$

This quantity gives the rate at which susceptible rodents become infected at time *t*.

Cumulative incidence ($I_{c}$).

This metric gives the total number of new infections generated over the full analysis horizon. It represents the absolute infection burden accumulated by the rodent population:

(15) $$I_{c}=\int_{0}^{t_{f}}I\left(\tau \right)\;d\tau =\int_{0}^{t_{f}}\beta_{0}\frac{S(\tau )I(\tau )}{K(F(\tau ))}\;d\tau .$$

Cumulative incidence per rodent-time (${\tilde{I}}_{c}$).

This normalized metric expresses accumulated new infections relative to the total rodent-time observed during the simulation:

(16) $${\tilde{I}}_{c}=\frac{I_{c}}{\int_{0}^{t_{f}}N\left(\tau \right)\;d\tau }.$$

This normalization supports comparisons among scenarios with different population sizes and habitat-mediated carrying capacities.

Average incidence rate ($\bar{I}$).

This metric gives the mean rate at which new infections arise over the full analysis horizon:

(17) $$\bar{I}=\frac{1}{t_{f}}\int_{0}^{t_{f}}I\left(\tau \right)\;d\tau .$$

It provides a time-averaged measure of transmission intensity and reduces the influence of short-term fluctuations.

Average infected load relative to ecological capacity (${\bar{P}}_{K}$).

We define

(18) $$P_{K}\left(t\right)=\frac{I(t)}{K(F(t))}$$

as infected load relative to ecological capacity. This quantity is not epidemiological prevalence and is not constrained to [0,1]. Its time average is

(19) $${\bar{P}}_{K}=\frac{1}{t_{f}}\int_{0}^{t_{f}}P_{K}\left(\tau \right)\;d\tau .$$

This indicator measures the mean crowding load of infected rodents relative to the ecological capacity provided by available habitat. Values above one indicate that the infected class transiently exceeds the instantaneous ecological capacity represented by K(F).

To evaluate threshold behavior after transients, we define the final analysis window as

(20) $$t_{*}=\max\left\{t_{0}+\varepsilon ,\;t_{f}-\varphi \right\},$$

where ε is the discarded transient time and φ is the length of the retrospective averaging window.

Average effective reproduction number (${\bar{R}}_{e}$).

This metric gives mean transmission potential over the final analysis window:

(21) $${\bar{R}}_{e}=\frac{1}{t_{f}-t_{*}}\int_{t_{*}}^{t_{f}}R_{e}\left(t\right)\;dt.$$

Values above one indicate that infection can expand on average during the final window. Values below one indicate a tendency toward decline.

Peak effective reproduction number ($R_{e}^{\max}$).

This metric records the strongest transmission episode during the final analysis window:

(22) $$R_{e}^{\max}=\max_{t\in [t_{*},t_{f}]}\left\{R_{e}\left(t\right)\right\}.$$

It identifies short periods of supercritical transmission that may occur even when the average effective reproduction number remains below one.

Time in the supercritical regime (Φ).

This metric measures the fraction of the final analysis window during which $R_{e}\left(t\right)>1$:

(23) $$\Phi =\frac{1}{t_{f}-t_{*}}\int_{t_{*}}^{t_{f}}1_{\left\{R_{e}\left(t\right)>1\right\}}\;dt,\;0\leq \Phi \leq 1.$$

This indicator distinguishes brief threshold crossings from sustained periods of epidemic expansion.

Together, these metrics separate cumulative infection burden, burden per rodent-time, infected crowding load, average transmission potential, peak supercriticality, and persistence above the epidemic threshold.

### 2.5. Sensitivity Analysis and Numerical Exploration

We use two complementary analyses. First, we compute normalized sensitivity indices for $R_{0}$ with respect to $\theta \in \{\beta_{0},b_{r},d,\gamma \}$:

(24) $$S_{\theta }^{R_{0}}=\frac{\partial \ln R_{0}}{\partial \ln\theta }=\frac{\theta }{R_{0}}\frac{\partial R_{0}}{\partial \theta }.$$

Second, we explore the (α,q) plane to quantify how restoration and degradation affect $I_{c}$, ${\tilde{I}}_{c}$, $\bar{I}$, ${\bar{P}}_{K}$, ${\bar{R}}_{e}$, $R_{e}^{\max}$, and Φ. For each parameter pair, system (8) is integrated over $t\in [0,t_{f}]$, and metrics are computed from the simulated trajectory. Full details of the numerical methods and the script that reproduces all figures are given in the Supplementary Material.

## 3. Results

**Figure 2:**
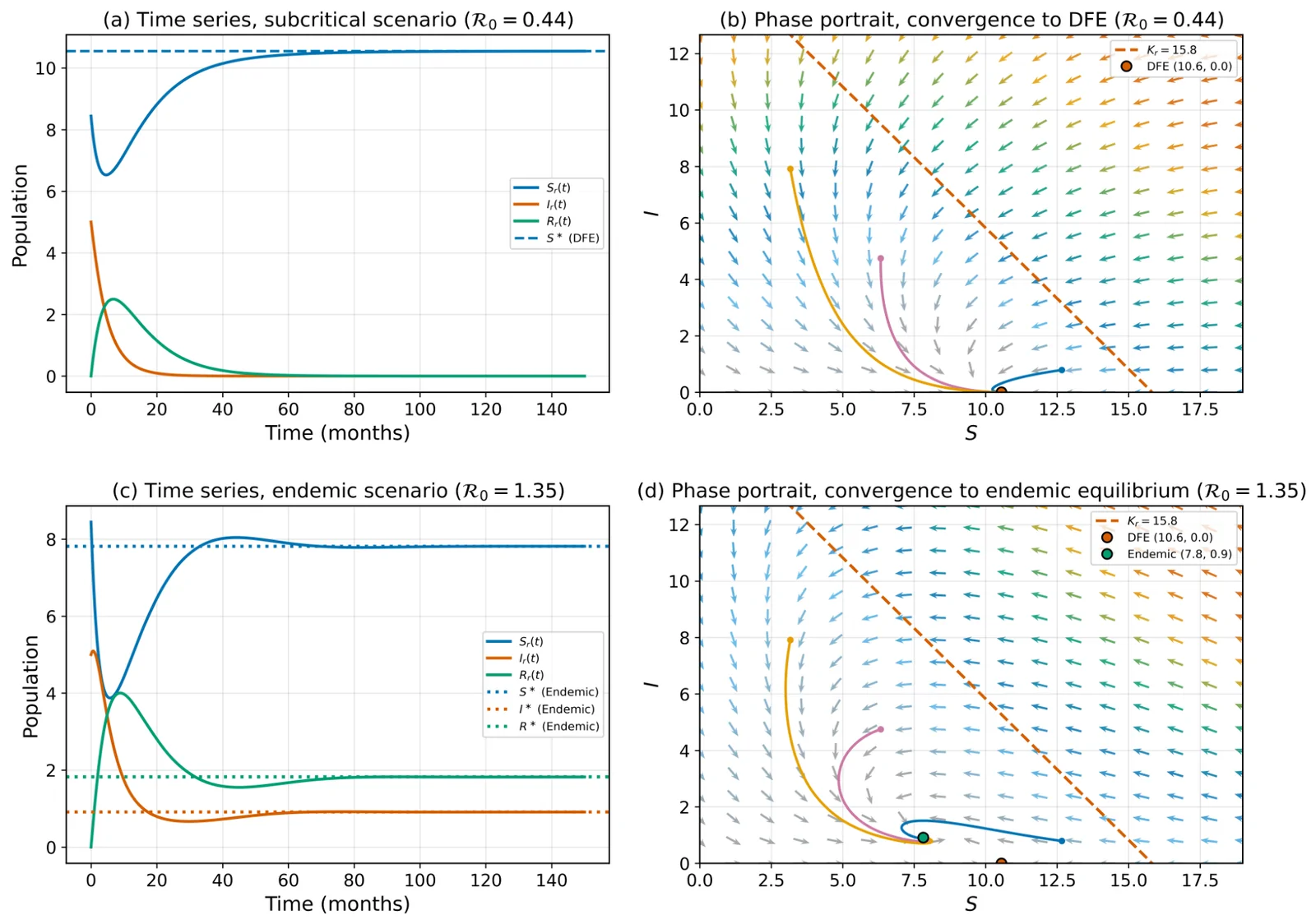
Contrast between disease-free and endemic dynamics in the coupled eco-epidemiological system. Time series show susceptible, infected, and removed rodents. Panels (**a**,**c**) display trajectories for subcritical and supercritical scenarios, respectively; panels (**b**,**d**) show the corresponding *S*–*I* phase portraits. In panels (**b**,**d**), the coloured arrows depict the direction field of the *S*–*I* subsystem, with colour encoding the local speed of the flow (blue slow, yellow to orange fast); the solid coloured curves are representative trajectories from different initial conditions; the dashed line marks the rodent carrying-capacity constraint; and the filled circles indicate the disease-free equilibrium in panel (**b**) and the endemic equilibrium in panel (**d**). All parameters are fixed at their baseline values except $\beta_{0}$, which is changed to place the system below or above the epidemic threshold. For the disease-free scenario, $\beta_{0}=0.198$ gives $R_{0}=0.44$; for the endemic scenario, $\beta_{0}=0.6075$ gives $R_{0}=1.35$. The remaining parameters are $b_{r}=0.30$, d=0.10, γ=0.20, $K_{F}=1.0$, $K_{r}=20$, $r_{F}=0.001$, q=0.010, and α=0.40.

The analytical results show the standard epidemic threshold structure. When $R_{0}<1$, the disease-free equilibrium $E_{0}$ is locally asymptotically stable. When $R_{0}>1$, the endemic equilibrium $E_{1}$ exists uniquely and is locally stable. The autonomous habitat equation yields the same habitat equilibrium in both epidemiological states, but transient habitat dynamics still affect incidence and $R_{e}\left(t\right)$ through K(F). Figure 2 contrasts representative subcritical and supercritical trajectories together with their phase portraits.

**Figure 3:**
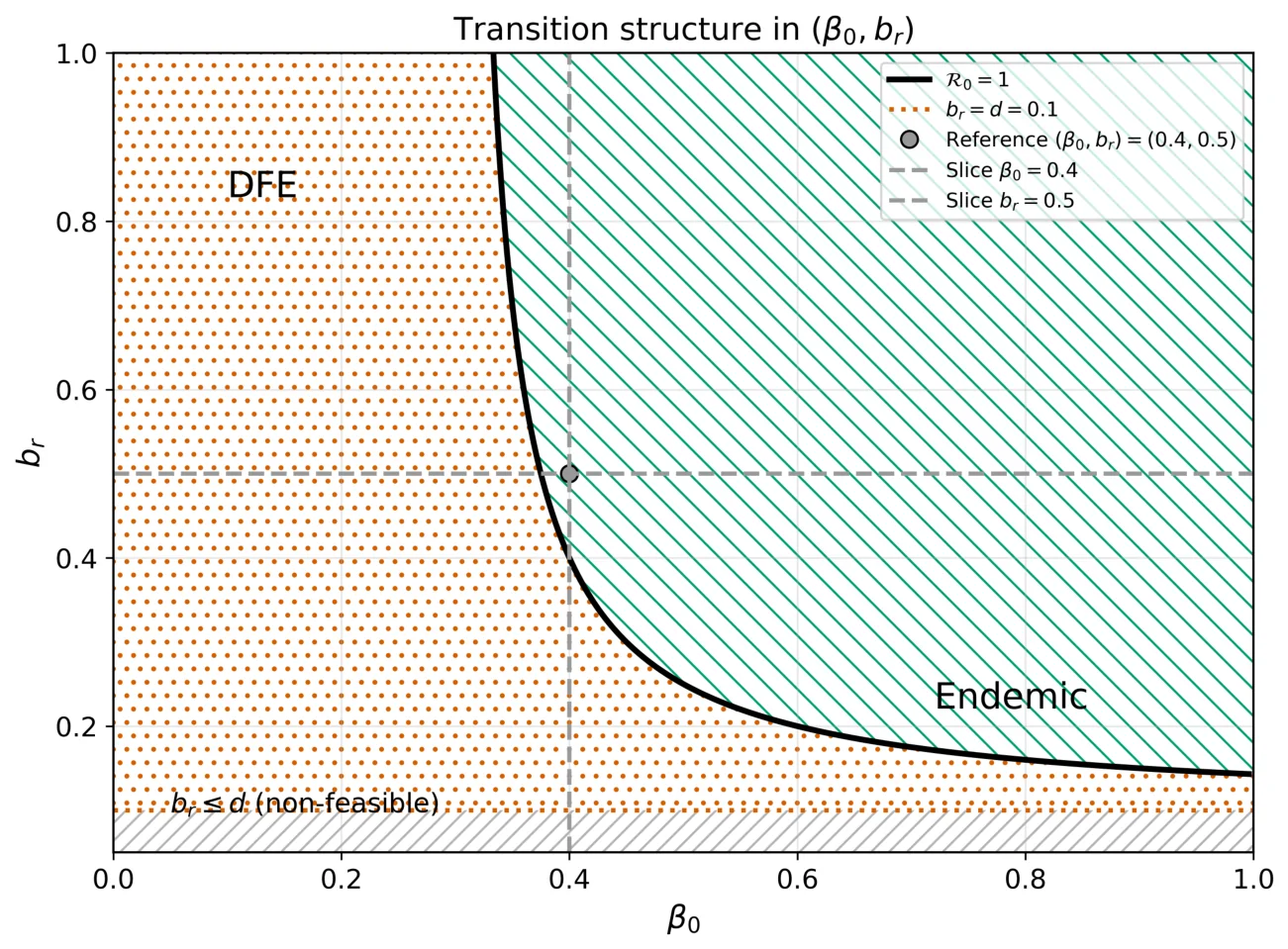
Threshold structure in the $\left(\beta_{0},b_{r}\right)$ plane. The curve $R_{0}=1$ separates the disease-free region from the endemic region. The band $b_{r}\leq d$ is biologically non-feasible because no positive rodent demographic equilibrium exists. The transition across the threshold is forward.

The threshold $R_{0}=1$ corresponds to a forward transcritical bifurcation. We use this result only to confirm that the deterministic model loses the endemic equilibrium when $R_{0}$ falls below unity. Figure 3 shows the resulting threshold structure, where the curve separates the disease-free and endemic regions. Appendix C retains the stability calculations.

Normalized sensitivity indices of $R_{0}$ are

(25) $$S_{\beta_{0}}^{R_{0}}=1,\;S_{b_{r}}^{R_{0}}=\frac{d}{b_{r}-d}>0,\;S_{d}^{R_{0}}=-\left(\frac{b_{r}}{b_{r}-d}+\frac{d}{d+\gamma }\right)<0,\;S_{\gamma }^{R_{0}}=-\frac{\gamma }{d+\gamma }<0.$$

**Figure 4:**
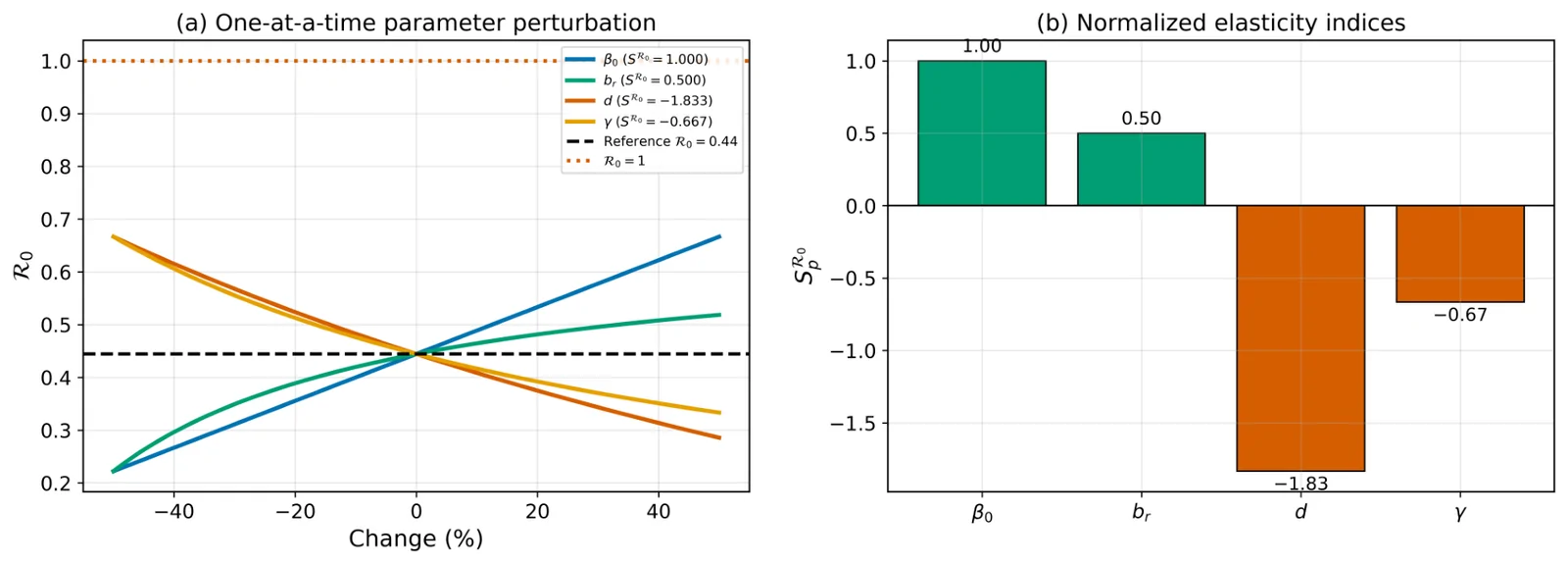
Sensitivity analysis of the basic reproduction number $R_{0}$. Panel (**a**) shows the response of $R_{0}$ to percentage changes in each parameter. Panel (**b**) shows normalized elasticity indices.

The unit elasticity of $\beta_{0}$ shows that $R_{0}$ scales linearly with transmission. Sensitivity to $b_{r}$ increases as $b_{r}\to d^{+}$, which highlights the role of positive demographic growth. Mortality and removal reduce $R_{0}$. Figure 4 displays these sensitivity and elasticity results.

### 3.1. Epidemiology Dependent on Habitat Cover

Numerical experiments in the (α,q) plane reveal a trade-off between absolute infection burden and crowding-mediated risk. High restoration and low degradation allow the reservoir population to approach higher carrying capacities K(F), increasing absolute incidence. High degradation without restoration produces smaller host populations but higher infected-load-to-capacity ratios because K(F) contracts.

**Figure 5:**
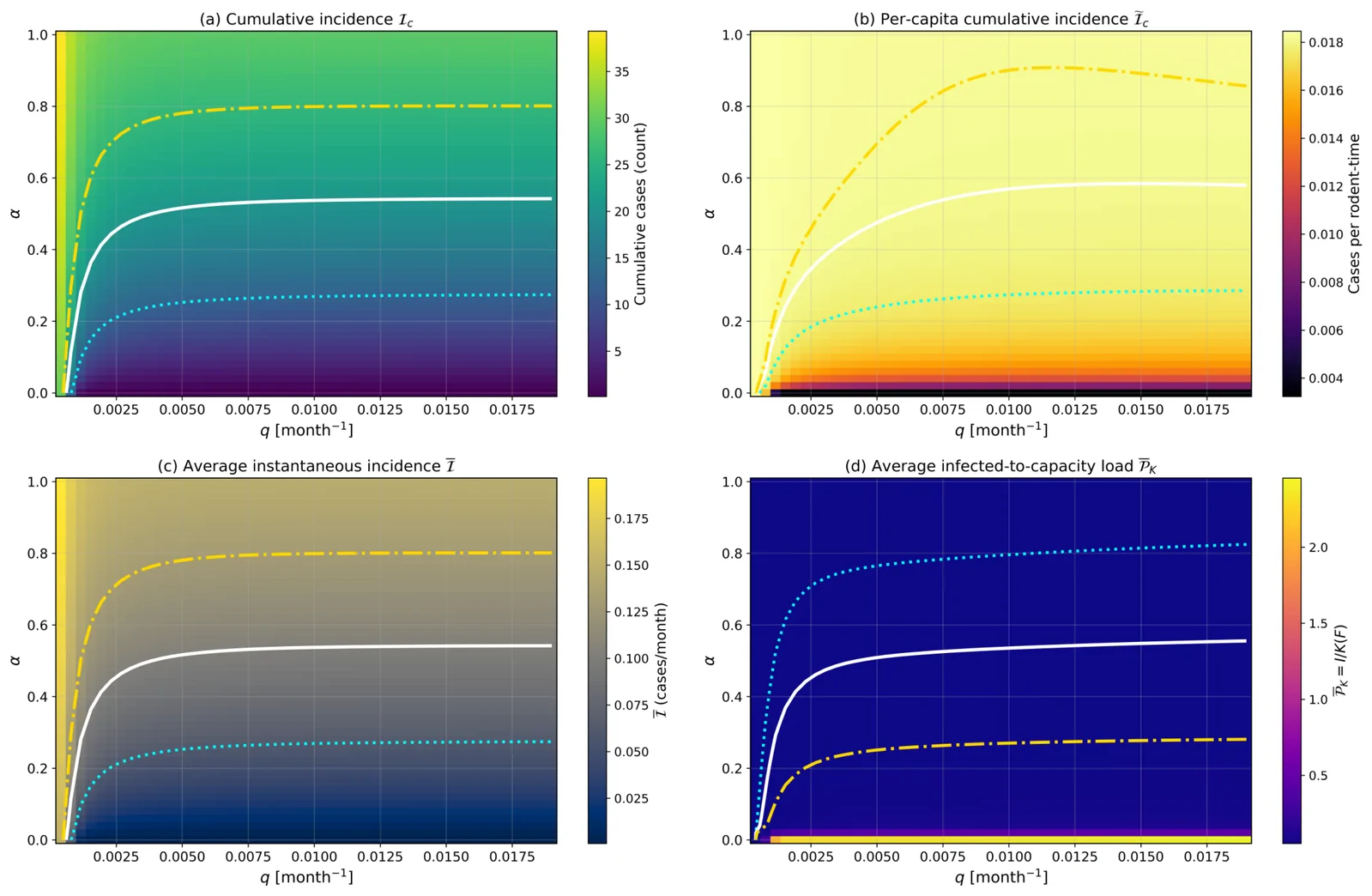
Sensitivity maps of epidemiological metrics in the habitat-management space (α,q). Maps show how restoration p=αq and degradation *q* modulate: (**a**) cumulative incidence $I_{c}$, (**b**) per-capita cumulative incidence ${\tilde{I}}_{c}$, (**c**) average incidence rate $\bar{I}$, and (**d**) average infected load relative to ecological capacity ${\bar{P}}_{K}$. For all panels, $\beta_{0}=0.45$, $b_{r}=$0.50, d=0.10, and γ=0.20, giving $R_{0}=$ 1.20. Variation across the maps is driven by habitat-mediated changes in K(F), incidence, and relative infected load, not by changes in the equilibrium threshold $R_{0}$. In every panel, the three overlaid contour lines mark percentiles of the displayed metric: the teal dotted line is the 25th percentile (P25), the white solid line is the median (P50), and the yellow dash-dotted line is the 75th percentile (P75).

Cumulative incidence $I_{c}$ and average incidence rate $\bar{I}$ reach their maxima with high restoration and low degradation, specifically at (α,q)=(1.00,0.0004) with $I_{c}\approx 39.86$ and $\bar{I}\approx 0.19$ (Table 4). In contrast, the average infected load relative to ecological capacity ${\bar{P}}_{K}$ is maximized with high degradation and no restoration, at (α,q)=(0.00,0.0190) with ${\bar{P}}_{K}\approx$2.73.This value is not prevalence; it indicates that infected abundance transiently exceeds the instantaneous ecological capacity represented by K(F). Figure 5 maps these epidemiological metrics across the habitat-management space.

### 3.2. Effective Reproduction Number and Temporal Persistence of Risk

**Figure 6:**
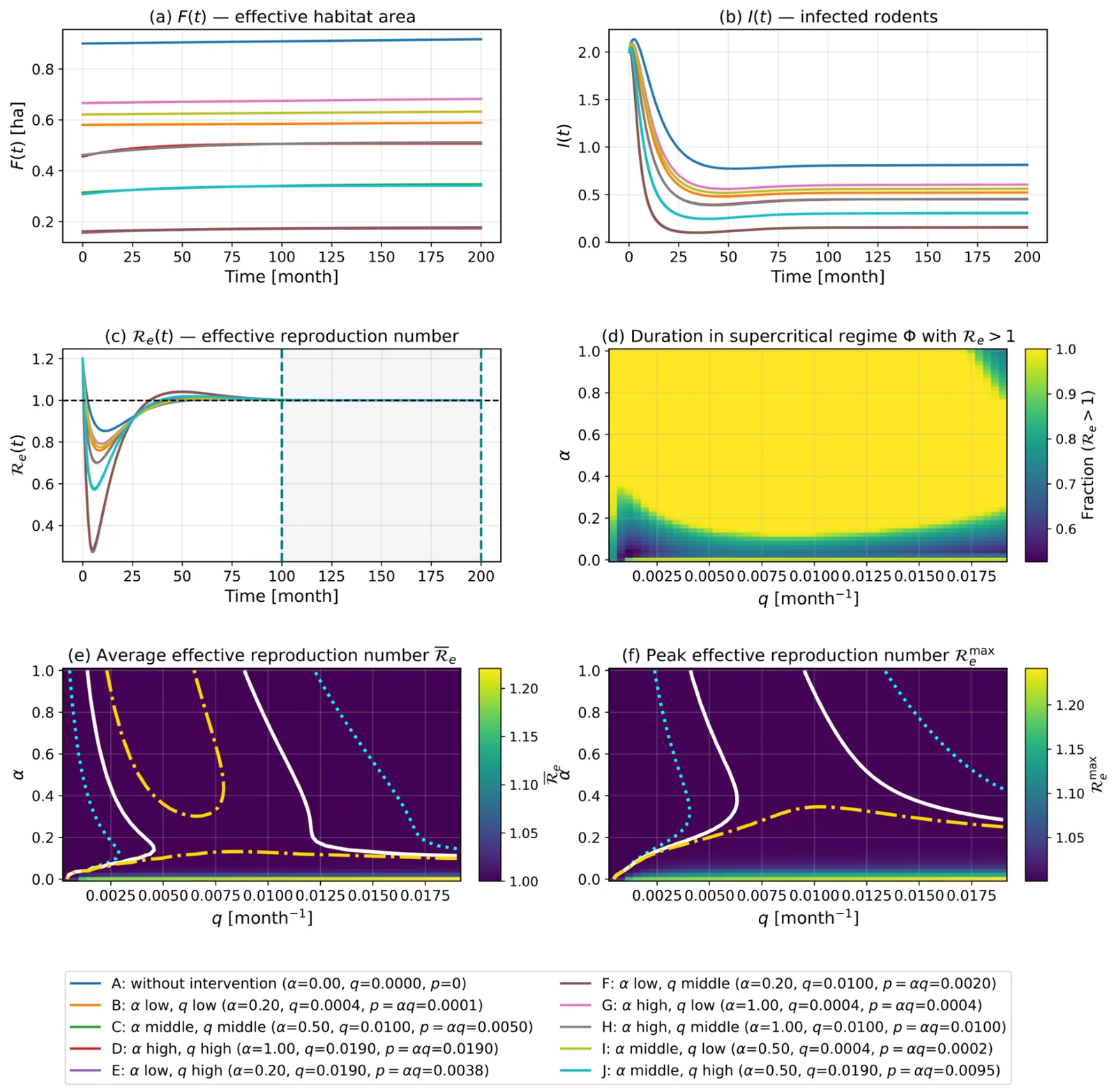
Temporal dynamics of the effective reproduction number $R_{e}\left(t\right)$ and epidemiological threshold indicators. Panels (**a**–**c**) show representative time series: (**a**) effective habitat F(t), (**b**) infected rodents I(t), and (**c**) $R_{e}\left(t\right)$, with vertical dashed lines delimiting the final analysis window $\left[t^{*},t_{f}\right]$. Panels (**d**–**f**) show heat maps in the (α,q) space: (**d**) the fraction of time in the supercritical regime, Φ; (**e**) the average effective reproduction number, ${\bar{R}}_{e}$; and (**f**) the maximum effective reproduction number, $R_{e}^{\max}$. Solid and dashed contour lines indicate median and interquartile contours, including P25 and P75. The basic reproduction number is fixed at $R_{0}=$ 1.20 in all scenarios;differences among curves and maps arise from the time-dependent ratio S(t)/K(F(t)) entering $R_{e}\left(t\right)$.

The effective reproduction number confirms that habitat acts through the trajectory-level ratio S(t)/K(F(t)). With high degradation and no restoration, both ${\bar{R}}_{e}$ and $R_{e}^{\max}$ reach their maxima at (α,q)=(0.00,0.0190), with ${\bar{R}}_{e}\approx 1.24$ and $R_{e}^{\max}\approx 1.25$. These moderately supercritical values can sustain infection over extended windows. The duration in the supercritical regime reaches Φ=1 at (α,q)=(0.00,0.0012), indicating persistent transmission potential even with lower degradation. Figure 6 shows the temporal dynamics of the effective reproduction number and the associated supercriticality indicators.

## 4. Discussion

### 4.1. Mechanistic Interpretation of Viral Persistence and the Abundance–Load Trade-Off

The model separates two epidemiological scales. Restoration raises K(F) and can increase absolute infection burden by supporting a larger host population. Degradation lowers K(F) and can increase relative infected load and $R_{e}\left(t\right)$ by concentrating infected rodents within reduced ecological capacity. This distinction explains why some scenarios produce high absolute incidence but low crowding pressure, whereas others produce low abundance but high infected load relative to capacity.

The environmental transmission route through aerosolized excreta reinforces this interpretation. Models with direct and indirect transmission show that demographic and seasonal coupling can sustain infection at modest rodent densities, even when direct transmission alone would not maintain $R_{0}>1$ [26,50]. In our model, $\beta_{0}$ absorbs effective contacts and exposure to contaminated environments, whereas I/K(F) captures the concentration of infectious rodents relative to ecological capacity.

Although the model is not calibrated to a specific locality, its qualitative behavior is consistent with several stylized facts of the *O. longicaudatus*–ANDV system. First, long-term surveillance in Chile shows a persistent, largely asymptomatic infection in which seropositive animals continue to shed virus, with a fraction of viremic hosts excreting virus into the environment [32]; the recent isolation of a novel ANDV haplotype from a viremic, initially seronegative host in a family cluster likewise indicates prolonged infectious potential [31]. This justifies our removed class *R* as seropositive individuals with reduced but non-zero shedding rather than fully immune hosts. Second, reservoir seroprevalence tracks host abundance across the latitudinal range, and higher seroprevalence is associated with greater human infection pressure [32], consistent with the dependence of $R_{e}\left(t\right)$ on the ratio S(t)/K(F(t)). Third, the highest Chilean seropositivity occurs in the Maule region, an area of agriculture and habitat modification where reservoir hosts can become dominant [32], agreeing with our result that degradation intensifies crowding-mediated infection pressure even when abundance is reduced. Fourth, populations with strong multi-annual fluctuations generate transient peaks of environmental contamination and infection [26], paralleling the supercritical windows (Φ, $R_{e}^{\max}$) our dynamics produce. These correspondences are qualitative: the model reproduces documented directions of association, not fitted magnitudes. Formal calibration against longitudinal seroprevalence and abundance series [32], together with environmental data, is the necessary next step toward locality-specific forecasting.

Gender-structured hantavirus models provide a related mechanism: heterogeneous aggressive contacts among territorial males can maintain transmission in fragmented populations [51]. Our model uses a different mechanism. Habitat contraction increases S(t)/K(F(t)) and thereby raises $R_{e}\left(t\right)$ through ecological crowding rather than sex structure.

### 4.2. Climate Forcing and Model Scope

The present model treats the demographic and transmission parameters as constants, which isolates the habitat-crowding mechanism but sets aside the meteorological and hydroclimatic oscillations that shape the demography of *O. longicaudatus* and the epidemiology of ANDV in temperate South American ecosystems. Clarifying how these drivers would enter the model delimits the scope of the present results and indicates how the framework extends without structural modification. Climate-driven pulses of primary productivity—masting in bamboo (*Chusquea*) and southern beech (*Nothofagus*), and precipitation anomalies linked to the El Niño–Southern Oscillation (ENSO)—modulate resource availability independently of physical habitat cover [21,32]. Such forcing would enter through the birth rate $b_{r}$ and the habitat regeneration rate $r_{F}$; likewise, because temperature and relative humidity govern how long deposited virus remains infectious, their effect would be carried by the effective coefficient $\beta_{0}$, which already aggregates direct and indirect exposure. In each case the driver would be represented as time variation of a parameter the model already contains, not as a new state variable.

This has a direct consequence for the threshold analysis. Because $R_{0}$ is evaluated at the disease-free equilibrium and is independent of $F^{*}$, seasonal or interannual forcing would leave the invasion threshold algebraically unchanged and act instead on the time-dependent quantities $R_{e}\left(t\right)$, $P_{K}\left(t\right)$, and the duration of the supercritical windows. Climatic variability would thus reshape the magnitude and timing of transient risk around the envelope characterized here, without reversing the direction of the habitat-crowding mechanism the model isolates.

This scope is consistent with the empirical literature. The distribution of *O. longicaudatus* in Argentine Patagonia is sensitive to temperature and precipitation and may contract with sustained climatic trends [52]; climate change is expected to influence hantaviruses chiefly through reservoir populations, with a plausible increase in ANDV infection frequency [53]; and higher precipitation promotes vegetation biomass and, through it, reservoir resources and outbreak risk [21]. Making $b_{r}$, $r_{F}$, and $\beta_{0}$ explicit functions of climatic drivers, and calibrating them to local climatological, abundance, and seroprevalence records within a One Health perspective [54], is an identifiable next step rather than a structural revision.

### 4.3. Habitat-Mediated Spillover Interpretation

Although humans are not explicitly modeled, the reservoir-level indicators provide a mechanistic basis for interpreting habitat-mediated spillover risk. Human ANDV infection occurs mainly through exposure to aerosolized excreta from infected rodents, and human-to-human transmission has been documented only for ANDV under specific outbreak conditions [12,13,14]. Scenarios with sustained $R_{e}\left(t\right)>1$, high infected load relative to ecological capacity, or prolonged supercritical windows mark ecological states in which the reservoir can maintain elevated infection pressure. These states do not predict human cases directly. They identify periods and habitat conditions where surveillance of rodents, environmental contamination, and human–rodent contact should intensify. This interpretation follows land-conversion spillover frameworks in which habitat change modifies host carrying capacities, local densities, and contact opportunities before human cases become observable [41].

An explicit human compartment would address a different question, namely, human incidence, and would require spillover and exposure parameters that are not identifiable without local case, serological, or household-contact data. The relationship between reservoir infection and human incidence is itself governed by complex and incompletely characterized ecological processes [26,32,42]. We therefore retain the human dimension implicitly through reservoir-level indicators, which characterize ecological conditions preceding potential human exposure rather than serving as a substitute for a human transmission model. Explicit linkage to household-level exposure and human surveillance data lies outside the scope of the present study. This distinction is important because changes in reservoir-level infection pressure and host abundance need not translate into equivalent changes in human exposure risk.

This becomes particularly relevant from a One Health perspective. Restoration may reduce crowding by increasing ecological capacity, but it can also support larger reservoir populations and increase the absolute number of infected rodents. Degradation may reduce host abundance while increasing infected load relative to remaining habitat capacity. Habitat management can therefore shift risk between absolute reservoir burden and crowding-mediated transmission pressure. Rodent-borne virus reviews now emphasize that surveillance should integrate reservoir demography, synanthropy, environmental change, and human exposure rather than treating host abundance alone as a sufficient risk proxy [4,8,9]. Spillover prevention should combine habitat management with measures that reduce peridomestic exposure, including safe cleaning practices, rodent exclusion, environmental sanitation, and targeted surveillance in rural and peri-domestic interfaces.

### 4.4. Implications for One Health Surveillance and Zoonotic Risk Reduction

For rodent-borne virus surveillance, the model identifies habitat conditions under which reservoir infection pressure may remain high even when host abundance is low. This point has global relevance because rodent-borne zoonotic risk depends on population fluctuations, synanthropy, land-use change, and the proximity between reservoir habitats and human activity [4,8,9]. In habitats with high degradation and low restoration, infection can persist with modest host abundance and sustained supercritical windows. Human risk in these settings likely depends on indirect exposure to aerosolized excreta in barns, warehouses, rural dwellings, and peri-domestic environments, a setting consistent with studies of peridomestic hantavirus transmission and small-mammal assemblages near human cases [32,50]. Interventions should reduce effective transmission through rodent exclusion, safe cleaning protocols, peridomestic sanitation, and community education rather than relying only on host reduction. In the model, these interventions correspond to reductions in $\beta_{0}$.

In habitats with high restoration and low degradation, larger reservoir populations increase the potential for human–rodent encounters and raise absolute infection burden in the reservoir. However, $R_{e}\left(t\right)$ remains subcritical in most simulated windows because greater ecological capacity reduces crowding. Restoration should therefore be paired with zoning, longitudinal rodent surveillance, serological monitoring, and prevention in areas where human activity overlaps with reservoir habitat. At the global level, this result cautions against treating habitat restoration as a purely protective intervention for rodent-borne viruses. Restoration can reduce density-dependent transmission pressure while increasing reservoir biomass, so surveillance programs should monitor both abundance and infected load.

The framework is transferable to other reservoir–hantavirus systems, but this transfer operates through parameter recalibration rather than structural change. The habitat-mediated crowding mechanism is generic: any system in which habitat regulates carrying capacity and infected-host concentration modulates transmission shares the same structure, as documented for *Peromyscus maniculatus* and Sin Nombre virus, where fragmentation increases infection rates [18,19], and as applied to *Apodemus*-borne hemorrhagic fever with renal syndrome through recalibration of an analogous compartmental skeleton [42]. What changes across host species, viruses, and countries is the parameter set, not the equations. The sensitivity analysis of $R_{0}$ (Section 2.5, Equation (25), Figure 4) makes this precise: the invasion threshold is governed by the epidemiological parameters $\beta_{0}$, $b_{r}$, *d*, and γ, with unit elasticity in $\beta_{0}$ and increasing sensitivity to $b_{r}$ as $b_{r}\to d^{+}$. These are therefore the quantities that must be re-estimated first when the model is moved to a new reservoir–virus system, whereas the algebraic form of the threshold is invariant. Because $R_{0}$ is independent of the equilibrium habitat state $F^{*}$, the epidemiological threshold is structurally separable from the habitat submodel: environmental and country-specific conditions enter through recalibration of the habitat parameters ($r_{F}$, *q*, α, $K_{F}$, $K_{r}$), which reshape the transient, habitat-dependent quantities $R_{e}\left(t\right)$, $P_{K}$, and the supercritical windows, but leave the equilibrium invasion threshold unchanged. Transferring the model thus requires recalibrating the epidemiological parameters for the invasion threshold and the habitat parameters for the transient regime, without altering the compartmental structure. Local seroprevalence, abundance, and habitat data for the target system [32] would supply these values.

This study provides a theoretical and mechanistic analysis, not a calibrated forecast for a specific locality. Parameter values were chosen from biologically plausible ranges reported for hantavirus reservoir systems and *O. longicaudatus* ecology, but the model was not fitted to seroprevalence or abundance time series from Chile or Argentina. The model also does not include a human compartment, explicit environmental viral persistence, or spatially resolved human–rodent contact. The numerical maps should therefore be read as qualitative threshold surfaces that identify reservoir-level mechanisms and risk regimes. Operational forecasting will require calibration with longitudinal rodent abundance, habitat-cover, environmental contamination, serological, and human-exposure data. A useful next step would link this reservoir model to spatial exposure layers, household-level prevention data, and human surveillance, so that ecological pressure in the reservoir can be evaluated against observed HCPS risk.

## 5. Conclusions

This study links habitat dynamics, reservoir demography, and ANDV transmission in a mechanistic eco-epidemiological framework for *O. longicaudatus*. The model separates absolute infection burden from crowding-mediated infection pressure. Restoration increases ecological capacity and can raise cumulative infection burden by supporting a larger reservoir population. Degradation reduces host abundance but can increase the infected-load-to-capacity ratio $P_{K}=I/K\left(F\right)$ and the effective reproduction number $R_{e}\left(t\right)$ through ecological crowding. The basic reproduction number $R_{0}$ does not depend on $F^{*}$ because susceptible abundance scales with carrying capacity at the disease-free equilibrium, whereas transient risk indicators depend on S(t)/K(F(t)).

This distinction has direct implications for rodent-borne virus surveillance. Low host abundance does not necessarily imply low transmission pressure, and high reservoir abundance does not necessarily imply high crowding-driven risk. Surveillance programs should therefore track abundance, serological or molecular infection indicators, habitat condition, and human–rodent exposure in the same monitoring design. For ANDV, this integrated view can help identify periods when habitat degradation, restoration, or peridomestic disturbance creates conditions for elevated reservoir infection pressure before human cases appear. Habitat restoration, degradation control, rodent surveillance, and exposure reduction should be evaluated together at human–rodent interfaces, with local calibration before operational forecasting.   

## Figures and Tables

**Table 1:** State variables of the eco-epidemiological model. Units: [ind] denotes individual rodents and [ha] denotes hectares.

Variable	Description	Unit
*N*	Total rodent population abundance, N=S+I+R.	[ind]
*S*	Susceptible rodents.	[ind]
*I*	Infected rodents, infectious and shedding virus.	[ind]
*R*	Removed rodents, interpreted as seropositive individuals with negligible or reduced shedding.	[ind]
*F*	Effective habitat: area-equivalent amount of usable habitat resources within the focal landscape.	[ha]
K(F)	Instantaneous carrying capacity modulated by habitat, $K\left(F\right)=\left(K_{r}/K_{F}\right)F$.	[ind]

**Table 3:** Equilibria and threshold conditions for system (8).

Equilibrium	Existence	Local Stability
Disease-free equilibrium $E_{0}$	$b_{r}>d$	stable if $R_{0}<1$
Endemic equilibrium $E_{1}$	$b_{r}>d$ and $R_{0}>1$	stable when it exists

**Table 4:** Maximum values by metric. Zeros in the column p=αq correspond to scenarios with α=0, that is, absence of compensatory restoration. They are structural values generated by the scenario design, not missing values.

Metric	Max Value	α	p=αq	*q*
Cumulative incidence $I_{c}$	39.86	1.00	0.0004	0.0004
Cumulative incidence per rodent-time ${\tilde{I}}_{c}$	0.018	0.00	0.0000	0.0004
Average incidence rate $\bar{I}$	0.19	1.00	0.0004	0.0004
Average infected load relative to ecological capacity ${\bar{P}}_{K}$	2.73	0.00	0.0000	0.0190
Average effective reproduction number ${\bar{R}}_{e}$	1.24	0.00	0.0000	0.0190
Peak effective reproduction number $R_{e}^{\max}$	1.25	0.00	0.0000	0.0190
Time in the supercritical regime Φ	1.00	0.00	0.0000	0.0012

## Data Availability

No new empirical data were generated in this study. The manuscript reports a mathematical model and numerical exploration. The simulation code (Python script) and parameter files are provided as Supplementary Material accompanying this submission.

## Supplementary Materials

The following supporting information can be downloaded at https://www.mdpi.com/article/10.3390/v18091024/s1: File S1: Numerical algorithm for the coupled habitat–SIR model (mathematical formulation, numerical integration methods, pseudocode, calculation of epidemiological metrics, and verification); File S2: andv_habitat_sir_model.py, the Python script (3.9+) that generates all figures in the main text.

## Abbreviations

The following abbreviations are used in this manuscript:

ANDV	Andes hantavirus
HCPS	Hantavirus cardiopulmonary syndrome
ODE	Ordinary differential equation
SIR	Susceptible–infected–removed

## Appendix A. Qualitative Properties of the Model

For nonnegative initial conditions with $0<F\left(0\right)\leq K_{F}$, system (8) has a unique solution that remains in the biologically feasible region. The habitat equation is scalar and locally Lipschitz on $\left(0,K_{F}\right]$. At $F=K_{F}$, $\dot{F}=-qK_{F}\leq 0$, so the upper boundary is inward pointing. If p>0, then $\dot{F}\to pK_{F}>0$ as $F\to 0^{+}$; if p=0, the no-restoration case requires $r_{F}>q$ for a positive habitat equilibrium. Thus trajectories with positive initial habitat remain positive in the biologically relevant parameter regime.

For the epidemiological variables, the vector field satisfies $\dot{S}\geq 0$ at S=0, $\dot{I}\geq 0$ at I=0, and $\dot{R}\geq 0$ at R=0. Hence the nonnegative orthant is positively invariant. Since N=S+I+R satisfies

(A1) $$\frac{dN}{dt}=B_{r}\left(N,K\left(F\right)\right)-dN,$$

and $B_{r}\left(N,K\left(F\right)\right)=0$ whenever N>K(F) in the positive-part natality function, and N(t) remains bounded. Therefore K(F(t))>0, and the force of infection is well-defined along biologically admissible trajectories.

## Appendix B. Derivation of Reproduction Numbers

At the disease-free equilibrium $E_{0}=\left(F^{*},S_{0}^{*},0,0\right)$, the infected equation linearized in *I* is

(A2) $$\frac{dI}{dt}=\left(\beta_{0}\frac{S_{0}^{*}}{K(F^{*})}-\left(d+\gamma \right)\right)I.$$

Since $S_{0}^{*}=K\left(F^{*}\right)\left(1-d/b_{r}\right)$, the next-generation ratio is

(A3) $$R_{0}=\frac{\beta_{0}}{d+\gamma }\left(1-\frac{d}{b_{r}}\right).$$

Along trajectories, the same linearized infection-growth argument gives

(A4) $$R_{e}\left(t\right)=\frac{\beta_{0}}{d+\gamma }\frac{S(t)}{K(F(t))}.$$

## Appendix C. Equilibrium and Stability Calculations

The disease-free equilibrium is $E_{0}=\left(F^{*},K\left(F^{*}\right)\left(1-d/b_{r}\right),0,0\right)$, provided $b_{r}>d$. The endemic equilibrium satisfies $\dot{I}=0$ with I>0, which gives $S_{1}^{*}=\left(d+\gamma \right)K\left(F^{*}\right)/\beta_{0}$. Substitution into the demographic balance and $\dot{R}=0$ yields the expressions in (13). Positivity of $I_{1}^{*}$ is equivalent to $R_{0}>1$.

The Jacobian at $E_{0}$ has one infection eigenvalue

(A5) $$\lambda_{I}=\left(d+\gamma \right)\left(R_{0}-1\right).$$

Hence the disease-free equilibrium is locally asymptotically stable when $R_{0}<1$ and unstable when $R_{0}>1$. When $R_{0}>1$, the endemic equilibrium is unique. The exchange of stability at $R_{0}=1$, together with the positivity of the endemic branch for $R_{0}>1$, gives a forward transcritical bifurcation. The full Jacobian can be reconstructed directly from (8); it was omitted from the main text to avoid interrupting the biological interpretation of the threshold results.
