# Supplementary material for: Habitat-Mediated Spillover Risk of Andes Hantavirus in Oligoryzomys longicaudatus: A Mechanistic Eco-Epidemiological Model
Source: Viruses. 2026 Sep 15;18(9):1024. doi: 10.3390/v18091024 (PMC13611947; doi:10.3390/v18091024)
Supplement: Supplementary file 1 [file viruses-18-01024-s001.zip › File S1 Numerical algorithm for the coupled.pdf]

Supplementary Material: Numerical Algorithm for the Coupled Habitat-SIR Model

Habitat-Mediated Spillover Risk of Andes Hantavirus in *Oligoryzomys longicaudatus*: A Mechanistic Eco-Epidemiological Model

Hendrik Sulbaran-Pineda[1,2]*ORCID, **Fernando Córdova-Lepe**[1]*ORCID, **Luis Pastenes**[1]ORCID, **Juan Pablo Gutiérrez-Jara**[3]ORCID, **Beatriz Cancino-Faure**[4]ORCID

[1] Facultad de Ciencias Básicas, Universidad Católica del Maule, Talca 3480112, Chile
[2] Centro de Ecología, Instituto Venezolano de Investigaciones Científicas, Altos de Pipe 1204, Miranda, Venezuela
[3] Centro de Investigación de Estudios Avanzados del Maule, Universidad Católica del Maule, Talca 3480112, Chile
[4] Facultad de Medicina, Universidad Católica del Maule, Talca 3480112, Chile

Correspondence: hendrik.sulbaran@alumnos.ucm.cl

Abstract

This supplement describes the numerical implementation of the eco-epidemiological model for Andes hantavirus (ANDV) transmission in *Oligoryzomys longicaudatus* presented in the main manuscript. We provide the mathematical formulation, the numerical integration methods (adaptive LSODA and fixed-step fourth-order Runge-Kutta), the pseudocode, the calculation of epidemiological metrics, and implementation details. The Python code used to generate all figures is also provided as a separate file.

1 Model equations

The model consists of four ordinary differential equations (ODEs) for effective habitat $F(t)$, susceptible $S(t)$, infected $I(t)$, and removed $R(t)$ rodents:

$$\frac{dF}{dt} = r_F F\left(1 - \frac{F}{K_F}\right) + p(K_F - F) - qF, \quad p = \alpha q, \tag{1}$$

$$\frac{dS}{dt} = B_r(N, K(F)) - dS - \lambda(t)S, \tag{2}$$

$$\frac{dI}{dt} = \lambda(t)S - (d + \gamma)I, \tag{3}$$

$$\frac{dR}{dt} = \gamma I - dR, \tag{4}$$

with $N = S + I + R$, $K(F) = \frac{K_r}{K_F}F$, $B_r(N, K) = b_r N\left(1 - \frac{N}{K}\right)_+$, $\lambda(t) = \beta_0 \frac{I}{K(F)}$.

All parameters and state variables are defined in Tables 1 and 2 of the main manuscript. The positive-part operator is defined as $(x)_+ = \max\{x, 0\}$.

2 Numerical methods

The system is integrated forward in time using two complementary approaches, depending on the required output.

2.1 Trajectory-based simulations (Figures 2 and 6 time series)

We use the adaptive LSODA method as implemented in `scipy.integrate.solve_ivp` (Python). LSODA automatically switches between non-stiff (Adams) and stiff (BDF) methods, with relative tolerance 10^{-6} and absolute tolerance 10^{-8}. The maximum time step is limited to $(t_f - t_0)/800$ to capture transient dynamics accurately.

2.2 Parameter-grid maps (Figures 5 and 6 heatmaps)

A custom **fourth-order Runge–Kutta (RK4)** integrator is used with a fixed time step $\Delta t = (t_f - t_0)/(N_t - 1)$, where $N_t = 1201$ points over $t \in [0, 200]$ months. The RK4 method is vectorised over the 2D grid of parameters (α, q) for efficiency. This explicit scheme is sufficient because the system is non-stiff for the chosen parameter ranges, and the fixed step size ensures consistent output for computing scalar metrics.

At each time step, positivity of state variables is enforced by clipping: $F \leftarrow \max(F, \varepsilon)$, and $S, I, R \leftarrow \max(\cdot, 0)$ with $\varepsilon = 10^{-9}$. The positive-part operator in the birth term is implemented as `np.maximum(1 - N/K, 0.0)`.

3 Pseudocode

The following pseudocode describes the RK4 integration for a single scenario (fixed parameters). The full grid implementation extends this to arrays using NumPy.

```
Input: parameters theta = {r_F, K_F, alpha, q, b_r, d, gamma, beta_0,
    K_r}
time grid t[0..M] with step dt
initial conditions F0, S0, I0, R0
Output: time series F, S, I, R at each t_k

Set F <- F0, S <- S0, I <- I0, R <- R0
For k = 0 to M-1:
# Compute derivatives at current state
K_cur = (K_r/K_F) * max(F, eps)
N_cur = S + I + R
B_cur = b_r * N_cur * max(1 - N_cur / K_cur, 0)
lam_cur = beta_0 * I / K_cur

dF1 = r_F*F*(1 - F/K_F) + alpha*q*(K_F - F) - q*F
dS1 = B_cur - d*S - lam_cur*S
dI1 = lam_cur*S - (d+gamma)*I
dR1 = gamma*I - d*R

# RK4 stage 2 (using half-step)
F2 = F + 0.5*dt*dF1; S2 = S + 0.5*dt*dS1; I2 = I + 0.5*dt*dI1; R2 = R +
     0.5*dt*dR1
K2 = (K_r/K_F)*max(F2, eps); N2 = S2+I2+R2
B2 = b_r*N2*max(1 - N2/K2, 0)
lam2 = beta_0*I2/K2
dF2 = r_F*F2*(1-F2/K_F) + alpha*q*(K_F - F2) - q*F2
dS2 = B2 - d*S2 - lam2*S2
dI2 = lam2*S2 - (d+gamma)*I2
dR2 = gamma*I2 - d*R2

# RK4 stage 3
F3 = F + 0.5*dt*dF2; S3 = S + 0.5*dt*dS2; I3 = I + 0.5*dt*dI2; R3 = R +
     0.5*dt*dR2
K3 = (K_r/K_F)*max(F3, eps); N3 = S3+I3+R3
B3 = b_r*N3*max(1 - N3/K3, 0)
lam3 = beta_0*I3/K3
dF3 = r_F*F3*(1-F3/K_F) + alpha*q*(K_F - F3) - q*F3
dS3 = B3 - d*S3 - lam3*S3
dI3 = lam3*S3 - (d+gamma)*I3
dR3 = gamma*I3 - d*R3

# RK4 stage 4
F4 = F + dt*dF3; S4 = S + dt*dS3; I4 = I + dt*dI3; R4 = R + dt*dR3
K4 = (K_r/K_F)*max(F4, eps); N4 = S4+I4+R4
B4 = b_r*N4*max(1 - N4/K4, 0)
lam4 = beta_0*I4/K4
```

```
        dF4 = r_F*F4*(1-F4/K_F) + alpha*q*(K_F - F4) - q*F4
        dS4 = B4 - d*S4 - lam4*S4
        dI4 = lam4*S4 - (d+gamma)*I4
        dR4 = gamma*I4 - d*R4

        # Update and enforce positivity
        F <- max(F + (dt/6)*(dF1 + 2*dF2 + 2*dF3 + dF4), eps)
        S <- max(S + (dt/6)*(dS1 + 2*dS2 + 2*dS3 + dS4), 0)
        I <- max(I + (dt/6)*(dI1 + 2*dI2 + 2*dI3 + dI4), 0)
        R <- max(R + (dt/6)*(dR1 + 2*dR2 + 2*dR3 + dR4), 0)

        Store F, S, I, R at t[k+1]
        End For
```

Listing 1: Fixed-step RK4 integrator for one parameter set

4 Initial conditions

For each simulation, the initial effective habitat $F(0)$ is set to $0.9F^*$, where F^* is the positive equilibrium of the habitat equation (Eq. (4) in the main manuscript). The initial susceptible population is $S(0) = 0.9K(F^*)(1 - d/b_r)$ when $b_r > d$; otherwise it is set to a small positive number. Infecteds are initialised as $I(0) = 2$ individuals, and $R(0) = 0$. This choice avoids the trivial disease-free equilibrium and allows the system to approach its attractor.

For the grid scans in Figures 5 and 6, the same logic is applied independently for every pair (α, q) using the corresponding F^*.

5 Calculation of epidemiological metrics

All metrics defined in Section 2.5 of the main manuscript are computed from the numerical solution using the trapezoidal rule:

- **Cumulative incidence** $\mathcal{I}_c = \int_0^{t_f} \beta_0 \frac{SI}{K(F)}\, dt$ Approximated as $\sum_{k=0}^{M-1} \frac{1}{2}(\mathcal{I}(t_k) + \mathcal{I}(t_{k+1}))\Delta t$.
- **Average infected load relative to capacity** $\overline{\mathcal{P}}_K = \frac{1}{t_f} \int_0^{t_f} \frac{I(t)}{K(F(t))}\, dt$ Similarly computed with trapezoidal rule.
- **Effective reproduction number** $\mathcal{R}_e(t) = \frac{\beta_0}{d+\gamma} \frac{S(t)}{K(F(t))}$ Evaluated at each time point; the average $\overline{\mathcal{R}}_e$ and peak $\mathcal{R}_e^{\max}$ are taken over the final analysis window $[t_*, t_f]$ (where $t_* = t_f - 100$ months after discarding a burn-in of 50 months). The fraction Φ of time with $\mathcal{R}_e(t) > 1$ is obtained by linear interpolation of the indicator function.

6 Implementation details

- **Language and libraries:** Python 3.9+, with NumPy (≥1.20), SciPy (≥1.6), and Matplotlib (≥3.4).
- **Parallelisation:** The grid scans are vectorised using NumPy's array operations; each RK4 step updates entire 2D arrays of states simultaneously, avoiding explicit loops over parameter pairs. This reduces runtime to a few minutes on a standard workstation.
- **Handling of non-feasible parameters:** When $b_r \leq d$ or when the habitat equilibrium would be zero, the code sets the corresponding equilibrium to a small positive value ($\varepsilon = 10^{-9}$) to avoid division by zero in the force of infection.
- **Reproducibility:** All random seeds are fixed (where applicable). The script saves all generated figures as high-resolution PNG (600 dpi) and, optionally, PDF. A verification report (`figures_verification_report.`[illegible] is also produced, listing the maxima of all metrics across the parameter grid.
- **Code availability:** The complete Python script that generates all figures is provided as a separate file (`andv_habitat_sir_model.py`).

7 Verification

The numerical implementation was verified by:

1. Comparing the equilibrium solutions from the ODE integrator with the analytical expressions (Eqs. (4), (9)–(11) in the main manuscript) for a range of parameters; the relative error was always below 10^{-8}.

2. Checking conservation of total population when $\beta_0 = 0$ (no transmission) against the analytical solution of the logistic-type demographic equation.

3. Ensuring that the computed $\mathcal{R}_0$ from numerical simulation of the infection growth phase matched the formula $\mathcal{R}_0 = \frac{\beta_0}{d+\gamma}\left(1 - \frac{d}{b_r}\right)$ to within 0.1%.

8 How to cite this supplementary material

Sulbaran-Pineda, H., Córdova-Lepe, F., Pastenes, L., Gutiérrez-Jara, J. P., & Cancino-Faure, B. (2026). Numerical algorithm for the habitat-mediated ANDV transmission model. Supplementary Material accompanying the manuscript "Habitat-Mediated Spillover Risk of Andes Hantavirus in *Oligoryzomys longicaudatus*: A Mechanistic Eco-Epidemiological Model".

References

[1] Sulbaran-Pineda, H. et al. Habitat-Mediated Spillover Risk of Andes Hantavirus in *Oligoryzomys longicaudatus*: A Mechanistic Eco-Epidemiological Model. *Manuscript submitted for publication*, 2026.
